# Comment on “Efficacy of laparoscopic parenchyma-sparing hepatectomy using augmented reality navigation combined with fluorescence imaging for colorectal liver metastases: a retrospective cohort study using inverse probability treatment weighting analysis”

**DOI:** 10.1097/JS9.0000000000003281

**Published:** 2025-08-25

**Authors:** Zhou Binghao, Li Chao, Xiaohu Guo

**Affiliations:** a The Second Clinical Medical College of Lanzhou University, Lanzhou University, Lanzhou, China; b General Surgery Department, Institution, The Second Hospital & Clinical Medical School, Lanzhou University, Lanzhou, Gansu, China

*Dear Editor*,

I read with great interest the recent study by Zeng *et al*^[1]^ published in the *International Journal of Surgery*, which explores the efficacy of laparoscopic parenchyma-sparing hepatectomy using augmented reality navigation combined with fluorescence imaging for colorectal liver metastases. The study offers valuable insights that in laparoscopic PSH for CRLMs, ARN-FI may improve surgical efficiency and accuracy. Especially for deep-seated tumors, it has the potential to reduce blood loss and attain higher R0 resection rates. The results may help to develop guidelines for the treatment of CRLMs and thus improve the prognosis of patients. Despite the strengths of this study, several underlying concerns require further elucidation. This article complies with the TITAN guidelines and acknowledges that no artificial intelligence was used^[2]^.

First, the evaluation of surgical complexity has historically been conventionally dependent on surgeons’ subjective assessments, rendering it inherently susceptible to variations in operator experience levels. Notably, objective preoperative risk stratification remains imperative for optimizing patient safety and perioperative outcomes^[3,4]^.In the present study, no comparative analyses were provided regarding the application of ARN-FI across surgeons with varying levels of seniority (e.g., junior vs. attending surgeons). Additionally, while the Iwate stratification index is mentioned in Table 1, the article does not further investigate the potential relationship between risk stratification and treatment outcomes. The lack of these essential datasets limits the ability to comprehensively evaluate the generalizability of the tool across diverse clinical settings and levels of operator expertise, which may introduce confounding factors into the interpretation of results. Therefore, it is recommended that future studies include stratified evaluations of ARN-FI performance across different tiers of surgical experience, in conjunction with case classification based on the Iwate difficulty index, to better understand their combined impact on outcomes through multivariate analysis.

Furthermore, the authors examined the impact of molecular markers, including KRAS mutation status and dMMR status, as well as the distinction between wild-type and mutant CRLM (Colorectal Liver Metastases), on ARN-FI. The analysis demonstrated that mutant KRAS serves as an independent risk factor for both Recurrence-Free Survival (RFS) and Liver-specific Recurrence-Free Survival (liver RFS). While the study commendably incorporated several primary tumor biology-related variables potentially influencing prognosis within its defined scope, certain pertinent variables warrant consideration for a more comprehensive analysis. Notably, research by Margonis *et al* investigating metastatic colorectal liver cancer identified the V600E BRAF mutation as a significantly stronger negative prognostic indicator compared to KRAS mutations^[5]^. Inclusion of BRAF mutation detection and corresponding data analysis would substantially enhance the robustness and explanatory power of the study’s conclusions. Moreover, exploring the differential efficacy of ARN-FI across various molecular subtypes of CRLM (e.g., KRAS mutant, BRAF mutant, RAS/BRAF wild-type, dMMR vs. pMMR) would provide a more direct and nuanced understanding of the risk factors associated with KRAS mutations and their correlations with treatment outcomes.

Thirdly, the subgroup analysis focusing on deep-seated tumors suggested a survival benefit associated with the ARN-FI group. However, Figure 3D (Kaplan-Meier curve) in the original manuscript reveals no statistically significant difference in recurrence-free survival (RFS) between the two groups for this specific subgroup. This apparent discrepancy between the reported subgroup benefit and the graphical representation of liver-specific RFS necessitates further clarification from the authors. To resolve this inconsistency and provide a more robust interpretation of the findings, we recommend that the authors offer a detailed explanation within the discussion section and consider presenting supplementary data to substantiate the observed subgroup effect.

Fourth, while the article appropriately acknowledges the dependence of ICG metabolism on hepatic uptake and excretory function, it fails to evaluate the potential confounding influence of pre-existing liver pathologies, such as fatty liver disease or cirrhosis, on the efficacy and interpretation of fluorescence imaging. To comprehensively assess the reliability of ICG-guided resection boundaries in diverse patient populations, we strongly recommend incorporating an analysis examining the correlation between quantifiable markers of hepatic parenchymal disease (specifically, the degree of fibrosis or steatosis) and the observed false-positive rate associated with ICG fluorescence. Such an analysis would be crucial for delineating the limitations of this technique in patients with compromised liver function and enhancing its clinical applicability.

In conclusion, this study contributes to the literature on treatment guidelines for colorectal liver metastases (CRLM). In addition to the aforementioned limitations, as noted by the authors, the relatively small sample size and single-center study design may constrain the generalizability of the findings. Addressing the issues outlined above would further strengthen the scientific rigor and clinical applicability of this work.

## Data Availability

No data was generated during the writing of this study.

## References

[1] Zeng X Li X Lin W. Efficacy of laparoscopic parenchyma-sparing hepatectomy using augmented reality navigation combined with fluorescence imaging for colorectal liver metastases: a retrospective cohort study using inverse probability treatment weighting analysis. Int J Surg 2025;111:1749–59. 39715148 10.1097/JS9.0000000000002193

[2] Riaz AA Ginimol M Rasha R. Transparency in the Reporting of Artificial Intelligence – the TITAN Guideline. Prem J Sci 2025;2:100082.

[3] Barron JO Orabi D Moro A. Validation of the IWATE criteria as a laparoscopic liver resection difficulty score in a single North American cohort. Surg Endosc 2022;36:3601–09. 34031739 10.1007/s00464-021-08561-4

[4] Lv X Zhang L Yu X Yu H. The difficulty grade of laparoscopic hepatectomy for hepatocellular carcinoma correlates with long-term outcomes. Updates Surg 2023;75:881–88. 36773170 10.1007/s13304-023-01452-4 PMC10285016

[5] Margonis GA Buettner S Andreatos N. Association of BRAF mutations with survival and recurrence in surgically treated patients with metastatic colorectal liver cancer. JAMA Surg 2018;153:e180996. 29799910 10.1001/jamasurg.2018.0996 PMC6137519

